# A Retrospective Study Examining the Effectiveness of the Study of the Management of Blunt Chest Wall Trauma (STUMBL) Scoring Tool in Predicting Complications Following Rib Fractures

**DOI:** 10.7759/cureus.101872

**Published:** 2026-01-19

**Authors:** Amber Ahmed-Issap, Marko Raseta, Kajan Mahendran, Lakshmi Srinivasan, Shilajit Ghosh, Udo Abah

**Affiliations:** 1 Cardiothoracic Surgery, Royal Stoke University Hospital, Stoke-on-Trent, GBR; 2 Statistics, Erasmus University Medical Center, Rotterdam, NLD

**Keywords:** chest wall trauma, morbidity and mortality, prognostic tools, rib fractures, stumbl

## Abstract

Introduction

Blunt thoracic trauma is a significant burden on the United Kingdom’s National Health Service. A common scoring system utilised to predict complications and guide management is the STUMBL (Study of the Management of Blunt Chest Wall Trauma) tool. This study evaluates the utility of a STUMBL score of >11 in predicting complications.

Methods

A retrospective study of patients with rib fractures was conducted at the University Hospitals of North Midlands, a major trauma centre in England. The primary outcome was rib fracture-related complications, defined as the occurrence of ≥1 of the following: 90-day mortality, pulmonary complications (including infection, pleural effusion and empyema, haemothorax, pneumothorax, and pulmonary contusions), ICU admission, or a hospital stay of ≥7 days. The area under the receiver operating characteristic curve (AUROC), sensitivity, specificity, positive predictive value (PPV), and negative predictive value (NPV) were calculated for a STUMBL score >11. Optimal cut-off scores for complications and 90-day mortality were also assessed.

Results

Between 01/06/2021 and 12/09/2023, 438 patients were enrolled in this study. A total of 199 patients were admitted to the hospital. Morbidity and mortality from rib fractures were 37.2% and 9.3%, respectively. The AUROC for a STUMBL score of >11 for all complications was 0.57 (sensitivity = 0.25, specificity = 0.88, PPV = 0.78, NPV = 0.41). The optimal STUMBL score for predicting overall complications and 90-day mortality was 18.5 (AUROC = 0.63) and 17.5 (AUROC = 0.62), respectively.

Conclusion

The STUMBL score of >11 poorly predicts complications in our population, whereas a score of 18.5 is more predictive. Further research is needed to determine if additional factors can enhance the utility of the STUMBL tool.

## Introduction

The overall number of patients admitted to hospitals has increased by almost 50% from 1999 to 2019 in England and Wales [1]. Thoracic trauma has been reported to comprise 10-15% of all trauma-related hospital admissions globally, with rib fractures accounting for the most common type of clinically significant blunt thoracic trauma [2]. The nature of the injury and pre-existing condition of the patient result in the need for differing levels of care and specialist management.

Whilst mortality is challenging to estimate due to possible concomitant injuries and indirect mortality from sequelae, it has been estimated at approximately 10-13% [3]. With the possibility of life-threatening complications occurring, clinicians have a propensity to admit most patients with rib fractures [4]. However, hospital capacity to admit patients is at an all-time low [5], meaning that patients who are not expected to develop complications should not be admitted. To assist with this, risk-stratification tools have been designed to assess the severity of the rib fractures and the likelihood of complications following thoracic trauma.

Risk stratification tools

Prognostic tools theoretically assist clinicians in deciding which patients can be discharged safely and which should be admitted to the hospital [6]. Many risk stratification tools have been designed to support early decision-making in predicting adverse outcomes after rib fractures and reduce morbidity [7], such as the RibScore [8], Organ Injury Scale [9], and Chest Trauma Score [10]. These models use variables published in the literature to determine the complication rates following rib fractures [11], such as the number of rib fractures [12], age [13], presence of pulmonary injuries [14], or other organ damage [15]. Each of these tools has benefits and drawbacks and has different focuses, such as determining admission, grading the severity of the injury, or predicting complications. However, many of these models have fallen out of favour due to the score's complexity, lack of external validity, or poor predictability of complications [16].

The STUMBL score

A prognostic tool called the STUMBL (Study of the Management of Blunt Chest Wall Trauma) score is employed by most accident and emergency (A&E) departments in the UK to assist with timely decision-making [17,18]. The tool was developed to predict complications following blunt chest wall trauma [19]. It is calculated using five variables to predict complications: age, number of ribs fractured, presence of chronic lung disease, pre-injury anti-coagulation, and oxygen saturation levels. These risk factors were developed after research by Battle and colleagues [20], where risk factors were analysed from 174 patients who had suffered from rib fractures and were admitted to the hospital or discharged. Each risk factor is assigned a score: one point is added for every 10 years of age (starting from age 10), three points for each rib fracture, five points if the patient has a diagnosis of chronic lung disease, four points for pre-injury anticoagulation, and two points for each 5% decrease in oxygen saturation starting at 94%. The STUMBL score also helps to guide whether a patient should be discharged (total score = 0-11), admitted to the hospital (total score = 12-26), or receive input from the ICU team (total score ≥27), depending on the likelihood of developing complications.

Following the development of the STUMBL score, Battle and colleagues [19] went on to externally validate the prognostic tool to increase the confidence in its value to predict complications and therefore guide the management of patients suffering from rib fractures.

Gaps in current knowledge

Several studies have evaluated the scoring tool both within the UK and internationally, with varying conclusions regarding the effectiveness and validity of the STUMBL score in predicting complications [21-24]. Giamello and colleagues [23] have demonstrated that the STUMBL score is effective at predicting complications following isolated blunt chest wall trauma. However, the tool is not used consistently across A&E departments in the United Kingdom (UK), as some data indicate that the score is no better than an emergency physician’s clinical judgement and that the STUMBL score, in fact, results in more patients being admitted when it is unnecessary [22]. Additionally, Webb and colleagues [24] recently demonstrated that the STUMBL score is suboptimal in predicting complications in a study involving 300 patients, but was specific in its ability to predict ICU admissions. Mukerji and authors [21] also report that the score did not predict the risk of complications with high enough sensitivity for it to be recommended in the New Zealand population. Some groups have modified the scoring tool from its initial use of predicting complications to assessing the need for analgesia depending on the severity of the STUMBL score [25].

Despite the inconsistencies in the conclusions of the validation studies, the STUMBL score remains the most common scoring tool employed for predicting complications and, consequently, hospital admissions after rib fractures in UK A&E departments and is recommended by national guidelines within the UK [7]. Therefore, the purpose of this study was to conduct a retrospective analysis of the predictive value of the STUMBL score at a large major trauma centre and evaluate whether A&E clinicians are currently utilising this recommended tool to predict complications and admission.

Aims

The primary aim of this study is to determine if the STUMBL score accurately predicts patient complications after blunt thoracic trauma, including pulmonary complications, extended hospital stay (≥7 days), and 90-day mortality. Secondary outcomes that will be assessed are threefold: to establish whether the STUMBL tool is being used to determine admission following isolated blunt thoracic trauma in the A&E of a major trauma centre. Next, a comparison will be made to evaluate the accuracy of this scoring tool in predicting complications compared to a clinician's judgement only. Finally, this study will assess whether the STUMBL score is an appropriate tool to guide hospital admission or if further calibration work is required for optimal performance.

## Materials and methods

Study design and population

All patients with rib fractures at a single major trauma centre (University Hospitals of North Midlands) in England from 01/06/2021 to 12/09/2023 were included. Patient variables were extracted by a single researcher with experience in this field. Information was collected on the patient’s pre-injury demographic, health status, and comorbidities. Patients were initially identified by the hospital’s Information and Performance team from A&E department discharge letters with a discharge diagnosis of ‘rib fractures’ or its derivatives including ‘fracture of rib’, ‘fracture of rib closed’, ‘fracture of rib open’, ‘multiple fractures of ribs’, ‘multiple fractures of ribs closed’, ‘multiple fractures of ribs open’, ‘flail chest closed’ or ‘flail chest open’. These were in accordance with the International Classification of Diseases, 10th revision (ICD-10) principal codes of any blunt thoracic trauma [26]. Only patients with rib fractures without concomitant injuries or polytrauma patients and those aged over the age of 18 years were included in this study.

Exclusion criteria included missing data variables to calculate the STUMBL score, age <18 years old, patients with polytrauma, lack of imaging scans, ‘old’ rib fractures, and reports of rib fractures without the exact number of rib fractures specified.

The original reference study that developed and validated the STUMBL score was used to determine the sample size for this research [19]. It indicated that out of 274 patients, 161 (59%) experienced complications, whereas the validation cohort had a complication rate of 103 out of 237 patients (43%). Following the one in ten rule, this study required 50 events to examine the rule, since the STUMBL score comprises five predictive variables. Thus, the study needs 85 patients when considering the original population with complications at a rate of 59%, or 117 patients when considering the complication rate reported in the validation cohort (43%). Therefore, this research ensured that at least double this number of patients were analysed to account for possible variations in complication rates. Consequently, data from over 27 months of A&E attendances were examined.

Outcomes

Data were collected from electronic records and paper notes scanned into the hospital’s electronic patient records platform. Each patient was searched manually with recordings of patient demographics, comorbidities, smoking status, mechanism of injury, admission vital signs, imaging type (e.g., chest X-ray (CXR) or computed tomography (CT) scan), and review of documentation of the STUMBL score in the patient’s notes.

The STUMBL score for all patients with isolated blunt thoracic trauma was calculated using Table 1. Chronic lung disease was defined as a patient with a documented diagnosis of chronic obstructive pulmonary disease (COPD), asthma, interstitial lung disease (ILD), pulmonary fibrosis, or emphysema.

**Table 1:** Study of the Management of Blunt Chest Wall Trauma (STUMBL) score. The score is assigned depending on patient variables. The total risk score determines the probability of complications. Depending on the score, patients will be assigned to the discharge home group (total score ≤11), where they are given an advice leaflet and analgesia, admitted to the hospital for observation, given analgesia, and receive physiotherapy (total score 12-26), or advised to receive intensive care input (total score ≥27). Adapted from Battle et al. [19] under the Creative Commons licence.

Risk factor	Scoring
Age	1 point for every 10 years of age (starting age 10)
Number of rib fractures	3 points per rib fracture
Chronic lung disease	5 points
Pre-injury anticoagulation use	4 points
Oxygen saturation levels	2 points for each 5% decrease in O_2_ saturation, starting at 94%

The researcher was blinded to the score calculation until all the data had been extracted from the objective score components and outcomes without any opportunity for individual interpretation, reducing the risk of bias. If oxygen saturation (SpO2) was recorded as ‘normal’ on room air (RA) in the medical notes, patients were given a normal value if there was no evidence of chronic lung disease (score 0 representing 95-100%) or a score of 2 (corresponding to 90-94%) if a patient had recorded evidence of chronic lung disease. If the patient was admitted to the A&E department with pre-hospital treatment of supplemental oxygen already being given in the field with no prior recording of SpO2 on RA, patients with chronic lung disease were assigned a score of 4 (representing 85-89%) whereas patients with no signs of chronic lung disease were assigned a score of 2 (corresponding to 90-94%).

Patient records with missing data variables required to calculate the STUMBL score were excluded from the study. On records where the researcher was unsure or managing ambiguity, they consulted with the principal research team, who agreed on a total score. As the aim was to validate the STUMBL score in its original form, no adjustment for confounders was performed.

The primary outcome was to assess the validity of the STUMBL score for all complications of blunt thoracic trauma. This was initially defined by Battle and colleagues [19] as the development of one of the following complications in medical records or imaging evidence: mortality, all pulmonary complications (including pneumothorax, haemothorax, pulmonary contusions, pneumonia, and empyema (collection of pus in the pleural space)), ICU admission, and prolonged hospital stay (defined as ≥7 days). Evidence of a lower respiratory tract infection (LRTI) or pneumonia was confirmed through imaging or documented evidence of the infection.

These complications were also used in this study to allow direct comparison. Additionally, the following outcomes were also recorded from medical records or imaging evidence: hospital admission, total hospital length of stay (LoS), need for surgical management of the rib fractures (namely, rib fixation surgery), chest infection (both in- or out-of-hospital, recorded from general practitioner (GP) records by patient note documentation of evidence of infection on respiratory examination, prescription of antibiotics, or imaging evidence of infection), presence of pulmonary embolism (PE), need for re-admission if discharged after initial rib fracture diagnosis, 90-day mortality (either in-hospital or out-of-hospital), and admission speciality (medicine versus surgical department).

Statistical analysis

A variety of different statistical methods were used during this study. Categorical data were summarised via numbers and percentages, while continuous data were summarised via means and standard deviations (SD) or medians and interquartile ranges (IQR), subject to the outcome of the Kolmogorov-Smirnov normality test. Equally, group comparisons of categorical data were done by means of the Z test and F test, subject to the expected cell counts, while group comparisons in continuous data were done by either the t-test or Mann-Whitney U-test, subject to the outcome of the Kolmogorov-Smirnov normality test. Predictive performance measures were reported for several adverse outcomes based on the existing well-recognised cut-offs already in print. Moreover, the Youden Index was used to deduce the optimal cut-offs for the existing scores. All statistical analyses were performed in the R statistical software version 4.5.1 (R Foundation for Statistical Computing, Vienna, Austria; packages: pROC and Caret) [27].

Ethical approval

The study was approved as an audit by the hospital audit team and the Ethics Committee at the University of Edinburgh.

## Results

Characteristics of study subjects

**Figure 1:**
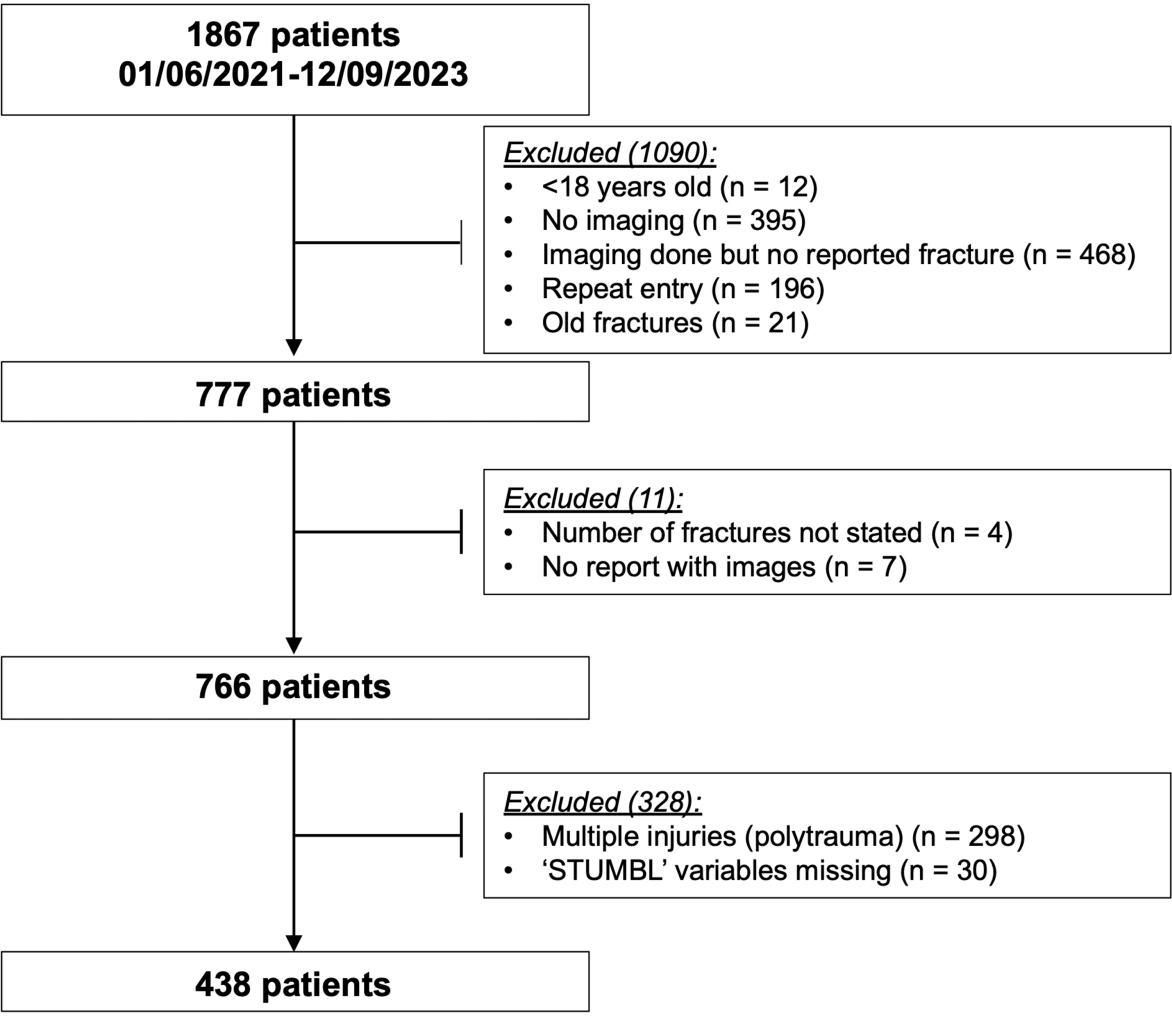
Diagram showing the flow of patients through the study. All patients with blunt thoracic trauma were identified between 01/06/2021 and 12/09/2023. Patients were excluded if they did not meet the inclusion criteria.

A total of 1867 patients were identified over a 27-month period. A total of 1428 patients were excluded who did not meet the inclusion criteria or had insufficient data to complete the STUMBL score. A total of 438 patients were included in this study (Figure 1).

Participant demographics are shown in Table 2. Median age of the participants was 69 years (IQR: 55.0-80.8), and 64.4% of the participants were male. Of the participants, 2.7% (n = 12) were admitted to the ICU with a mean ICU LoS of 8.5 days (SD±10.8). A CT scan was performed in 315 (71.9%) cases. A total of 65 (14.8%) patients were on anticoagulation, 138 (31.5%) patients had chronic lung disease, and 187 (42.7%) patients had hypertension. The majority of patients were non-smokers (n = 202, 46.1%), followed by ex-smokers (n = 151, 34.5%), with the fewest patients being current smokers (n = 84, 19.2%), who suffered rib fractures.

**Table 2:** Baseline demographics of study participants. IQR = interquartile range; BMI = body mass index; RTA = road traffic accident; A&E = accident & emergency; CT = computed tomography; CXR = chest X-ray; SpO2 = oxygen saturation; O2 = oxygen; HR = heart rate; SD = standard deviation; RR = respiratory rate; BP = blood pressure; mmHg = millimetres per mercury; AF = atrial fibrillation; HTN = hypertension; IVDU = intravenous drug user; RA = rheumatoid arthritis; OA = osteoarthritis; VTE = venous thromboembolism; LoS = length of stay; ICU = intensive care unit; STUMBL = Study of the Management of Blunt Chest Wall Trauma.

Participant demographics (n = 438)	
Age, median (IQR) (years)	69 (55, 80.8)
Number of rib fractures, median (IQR)	3 (1, 5)
BMI, median (IQR) (kg/m^2^)	26.4 (23.1-30)
Sex, n (%)	
Female	156 (35.6%)
Male	282 (64.4%)
Mechanism of injury, n (%)	
Fall from height	56 (12.8%)
Fall from standing	192 (43.8%)
Fall from sitting	19 (4.3%)
Fall down the stairs	62 (14.2%)
Direct chest trauma	10 (2.3%)
Kicked by an animal	4 (0.9%)
Assault	14 (3.2%)
Sporting accident	5 (1.1%)
RTA – car	29 (6.6%)
RTA – motorbike	17 (3.9%)
RTA – bicycle	10 (2.3%)
RTA – pedestrian	4 (0.9%)
Other – cancer	4 (0.9%)
Other – caused by dystonia	1 (0.2%)
Other – coughing excessively	7 (1.6%)
Other – crushed by an animal	1 (0.2%)
Other – not stated	3 (0.7%)
Highest level of imaging in A&E, n (%)	
CT scan	315 (71.9%)
CXR	123 (28.1%)
SpO_2_ (%), n (%)	
SpO_2_ 95-100	340 (77.6%)
SpO_2_ 90-94	43 (9.8%)
SpO_2_ 85-89	8 (1.8%)
SpO_2_ 80-84	1 (0.2%)
SpO_2_ 75-79	0 (0.0%)
SpO_2_ 70-74	1 (0.2%)
SpO_2_ on supplemental O_2_	45 (10.3%)
HR, mean ± SD (beats/minute)	81.8 ± 16.4
RR, median [IQR] (breaths/minute)	18 (16, 20)
Systolic BP, mean ± SD (mmHg)	143.1 ± 25.8
Smoking status, n (%)	
Non-smoker	202 (46.1%)
Ex-smoker	151 (34.5%)
Current smoker	84 (19.2%)
Comorbidities, n (%)	
Pre-injury anticoagulation	65 (14.8%)
Chronic lung disease	138 (31.5%)
Cardiac disease	123 (28.1%)
Hypercholesterolaemia/ hyperlipidaemia	108 (24.7%)
Type 2 diabetes	70 (16.0%)
AF	50 (11.4%)
HTN	187 (42.7%)
History of kidney disease	62 (14.2%)
History of cancer	59 (13.5%)
History of stroke	47 (10.7%)
Current or ex-IVDU	7 (1.6%)
RA	9 (2.1%)
OA	92 (21.0%)
History of alcohol excess	38 (8.7%)
History of VTE	27 (6.2%)
Dementia	33 (7.5%)
Parkinson’s disease	12 (2.7%)
Hospital admission, n (%)	199 (5.4%)
Hospital LoS, median (IQR) (days)	6 (2.0, 10.7)
Admission speciality, n (%)	
Medicine	94 (47.2%)
Surgery	105 (52.8%)
ICU admission, n (%)	12 (2.7%)
ICU LoS, mean ± SD (days)	8.5 ± 10.8
STUMBL score, median (IQR)	19 (13, 24)

Morbidity and mortality of rib fractures

**Figure 2:**
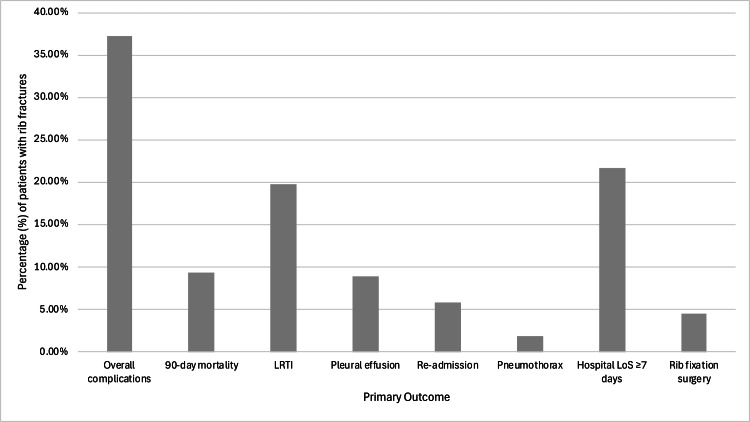
Morbidity and mortality of rib fractures. The percentage of patients who developed pulmonary complications, mortality, required re-admission after initial discharge, or rib fixation surgery is displayed. LoS = length of stay; LRTI = lower respiratory tract infections.

Of the patients, 37.2% (n = 163) developed complications (Figure 2). The most common complications were LRTIs (n = 87, 19.9%), followed by pleural effusions (n = 39, 8.9%). A total of 21.7% of patients had a prolonged hospital LoS.

STUMBL score validation

STUMBL scores were calculated from Table 1. Mean STUMBL score was 19.0 (SD±8.6). The risk score and corresponding risk of developing complications are shown in Table 3. Up to a STUMBL score of 30, the probability of complications with every cohort increased. However, the 26-30 (59.6%) and 31+ (57.8%) groups yielded a similar prediction of the probability of developing complications. In the patients who were admitted (n = 199, 45.4%), 190 (95.4%) patients had a STUMBL score of >11, and nine (4.5%) patients had a STUMBL score of 0-11. In the discharged population (n = 239, 54.6%), 79 (33.0%) patients had a score of ≤11, and 160 (63.9%) patients had a score of >11.

**Table 3:** Risk score and corresponding risk of developing complications (n = 438). Categories were developed as recommended by the initial development and validation study. STUMBL = Study of the Management of Blunt Chest Wall Trauma.

STUMBL score	Probability of complications (%)	Number of patients in each category, n (% of population)
1 – 10	21.5%	79 (18.0%)
11 – 15	29.2%	96 (21.9%)
16 – 20	32.6%	95 (21.7%)
21 – 25	42.3%	71 (16.2%)
26 – 30	59.6%	52 (11.9%)
31+	57.8%	45 (10.3%)

**Figure 3:**
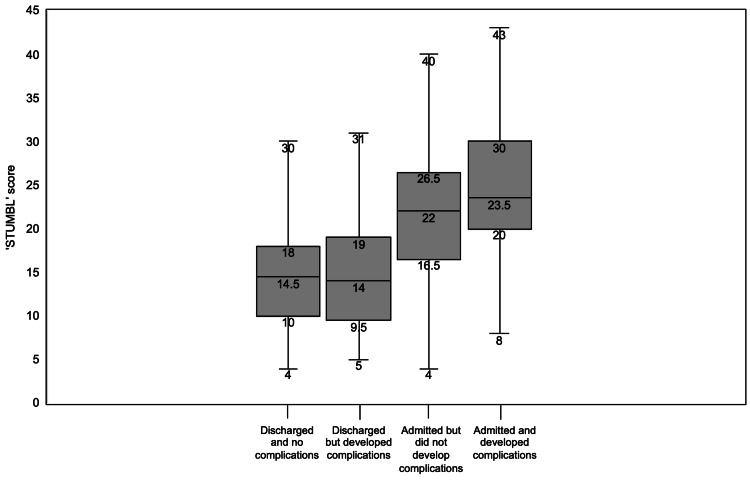
Box and whisker plots comparing the STUMBL score values. (Reading from left to right) for patients discharged from the emergency department with no complications developed, discharged but complications developed in the patient, admitted to the hospital but did not develop complications, and admitted to the hospital with eventual complication development. STUMBL = Study of the Management of Blunt Chest Wall Trauma.

The box and whisker plot in Figure 3 compares the STUMBL scores between patients discharged with no development of complications, discharged but developed complications, admitted with no complications, and admitted with the development of complications. Median STUMBL score for patients who were discharged and did not develop complications was 14.5, whilst those who were discharged but did develop complications did not have a significantly different score at 14.0. The median STUMBL score for patients who were admitted but did not develop complications was 22, whilst those who were admitted but did develop complications were 23.5.

**Figure 4:**
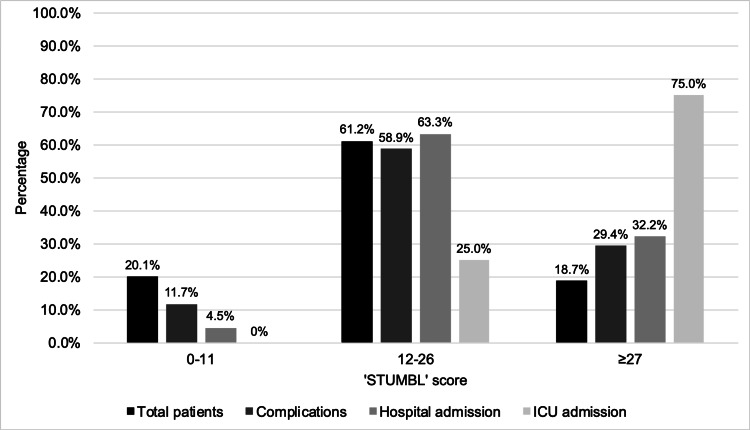
Complications and admission following blunt thoracic trauma categorised by the STUMBL score. A patient with a STUMBL score of 0-11 is recommended to be discharged, a patient with a STUMBL score of 12-26 is recommended to be admitted, and a score of ≥27 recommends ICU input. STUMBL = Study of the Management of Blunt Chest Wall Trauma.

Battle and colleagues [19] recommend that a STUMBL score of 0-11 means that the patient can be discharged due to a low risk of complications, a patient with a STUMBL score of 12-26 should be admitted to the hospital, and a score of ≥27 should have ICU input. Figure 4 shows the complications and hospital admissions categorised as recommended by Battle and colleagues [19]. A total of 88 (20.1%) patients had a score of 0-11, 268 (61.2%) patients had a score of 12-26, and 82 (18.7%) patients had a score of ≥27. In the 0-11, 12-26, and ≥27 STUMBL score categories, 11.7% (n = 19), 58.9% (n = 96), and 29.4% (n = 48) developed complications, respectively. Of hospital admissions, 4.5% (n = 9) were in the 0-11 group, 63.3% (n = 126) were in the 12-26 category, and 32.2% were in the ≥27 group. Mean hospital LoS in the 0-11, 12-26, and ≥27 groups were 2.8 days (SD ± 2.9), 7.09 days (SD ± 8.3), and 11.3 days (SD ± 10.3), respectively. The vast majority of patients who were admitted to the ICU were in the ≥27 group (n = 9, 75.0%), with only three patients (25.0%) in the 12-26 group admitted to the ICU. The ICU mean LoS was 10.5 days (SD ± 11.9) in the ≥27 group and 2.3 days (SD ± 1.5) in the 12-26 group.

**Figure 5:**
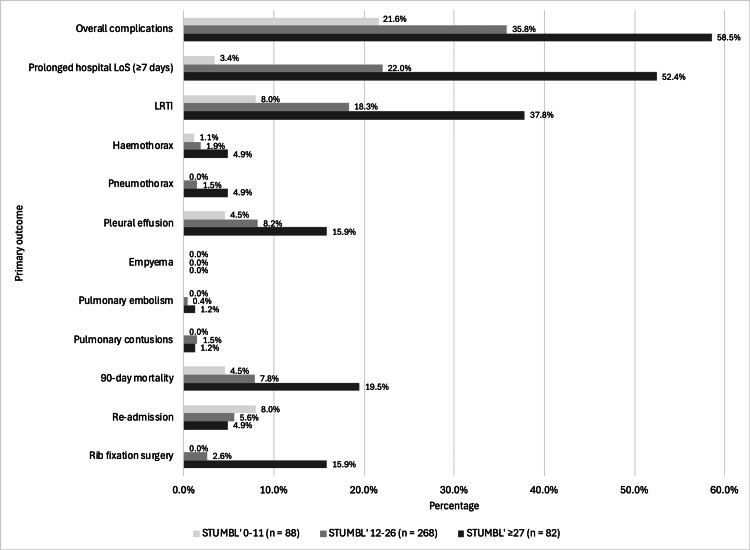
Complications categorised by the STUMBL score. A patient with a STUMBL score of 0-11 is recommended to be discharged, a patient with a STUMBL score of 12-26 is recommended to be admitted, and a score of ≥27 recommends ICU input. STUMBL = Study of the Management of Blunt Chest Wall Trauma; LoS = length of stay; LRTI = lower respiratory tract infections.

Complications were then analysed depending on the three STUMBL score categories (Figure 5). More patients with a score of ≥27 had a prolonged hospital LoS (n = 43, 52.4%) compared to the 12-26 group (n = 59, 22.0%) and the 0-11 group (n = 3, 3.4%). Of all the complications, LRTI was the most common in all groups, with most patients in the STUMBL ≥27 group developing an LRTI (n = 31, 37.8%). LRTI developed in 49 (18.3%) patients of the 12-26 group and seven (8.0%) in the 0-11 group. The 90-day mortality was highest in the STUMBL ≥27 group (n = 16, 19.5%). Interestingly, re-admission after initial discharge was highest in the 0-11 group (n = 4, 8.0%) and lowest in the STUMBL ≥27 group (n = 4, 4.9%), likely due to the majority of patients being initially admitted in the ≥27 group. Rib fixation surgery was most needed in the ≥27 group (n = 13, 15.9%).

A univariate analysis of the data shows that increasing STUMBL scores significantly predicted overall complications (Table 4). A STUMBL category of 12-26 increases the likelihood of developing complications by 1.7 times (OR = 1.72, 95% CI = 1.03-2.89, p < 0.05). A score of ≥27 increased the chances of developing complications by fourfold (OR = 4.40, 95% CI = 2.35-8.26, p < 0.001).

**Table 4:** Univariate analysis of the STUMBL score in developing overall complications with rib fractures. STUMBL = Study of the Management of Blunt Chest Wall Trauma.

STUMBL score range	Odds ratio	95% CI for the odds ratio	P-value
0-11 (reference category)	-	-	-
12-26	1.72	(1.03, 2.89)	0.04
≥27	4.40	(2.35, 8.26)	3.87 x 10^-6^

Comparison of the performance of A&E clinicians' decision to admit with a STUMBL score of >11 for predicting complications

Test characteristics for A&E clinicians' judgement for predicting the primary outcomes (overall complications) were: sensitivity = 0.65, specificity = 0.66, positive predictive value (PPV) = 0.53, and negative predictive value (NPV) = 0.76. For those admitted (n = 199), the mean hospital LoS was nine days (SD ± 9). A total of 106 (53.3%) admitted patients developed complications, 12 (6.0%) patients were admitted to the ICU, and 26 (13.1%) patients had a 90-day mortality. Of those discharged (n = 239), 57 (23.8%) developed complications, with LRTI being the most common of the overall complications in both the discharged and admitted cohorts (n = 18, 31.6%). Appendix shows the characteristics of patients who were discharged versus admitted to the hospital based on the clinician’s judgement.

Test characteristics for a STUMBL score of >11 for predicting the primary outcomes (overall complications) were based on the Youden Index: sensitivity = 0.25, specificity = 0.88, PPV = 0.78, and NPV = 0.41. Other outcomes were also measured using the Youden Index when the STUMBL score >11 (Table 5). For patients with a STUMBL score >11 (n = 350), 190 patients were admitted. Mean hospital LoS was 9.2 days (SD ± 9.4). The difference between the costings of A&E clinicians' judgement in being too cautious in admitting patients (admitted to the hospital but did not develop complications) and a STUMBL score >11 (recommended for admission due to high risk of complications), with these patients not developing complications, was not statistically significant (p = 0.60). The specificity of a STUMBL score of >11 in predicting ICU admission was highest (1.00). Further details of the predictability of the STUMBL score in predicting complications and the baseline characteristics categorised according to the STUMBL score of 0-11, 12-26, and >26 are presented in the Appendix.

**Table 5:** Performance measure of a STUMBL score of >11 in predicting overall complications, 90-day mortality, ICU admission, prolonged hospital LoS, and LRTI following rib fractures. PPV = positive predictive value; NPV = negative predictive value; ICU = intensive care unit; LoS = length of stay; prolonged hospital LoS defined as ≥7 days; LRTI = lower respiratory tract infection; STUMBL = Study of the Management of Blunt Chest Wall Trauma.

Performance measures	Overall complications	90-day mortality	LRTI	ICU admission	Prolonged hospital LoS
Accuracy	0.49	0.28	0.37	0.23	0.41
Sensitivity	0.25	0.21	0.23	0.21	0.25
Specificity	0.88	0.90	0.92	1.00	0.99
PPV	0.78	0.95	0.92	1.00	0.99
NPV	0.41	0.12	0.23	0.03	0.27

**Figure 6:**
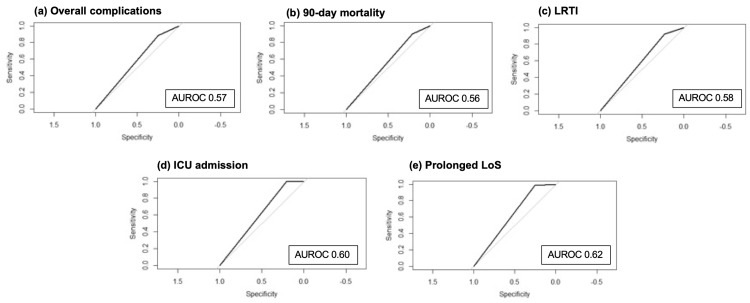
AUROC for a STUMBL score >11 on varying outcomes after blunt thoracic trauma. (a-f) AUROC for overall complications (a), 90-day mortality (b), development of lower respiratory tract infections (LRTIs) (c), intensive care unit (ICU) admission (d), and prolonged hospital length of stay (LoS) defined as ≥7 days (e). AUROC = area under the receiver operating characteristic curve; STUMBL = Study of the Management of Blunt Chest Wall Trauma.

The areas under the receiver operating characteristic curve (AUROC), measuring the effectiveness of a STUMBL score of >11 for overall complications, 90-day mortality, development of LRTI, ICU admission, and a prolonged hospital LoS, are shown in Figure 6.

Cut-off scores and risk thresholds

The optimal cut-off STUMBL score for predicting overall complications, 90-day mortality, ICU admission, and prolonged hospital LoS was based on the Youden Index (Table 6).

**Table 6:** Optimal STUMBL scores and corresponding performance measures for predicting overall complications, 90-day mortality, intensive care unit (ICU) admission, prolonged hospital length of stay (LoS) defined as ≥7 days, and surgery for rib fractures (rib fixation surgery) after blunt thoracic trauma. PPV = positive predictive value; NPV = negative predictive value; ICU = intensive care unit; LoS = length of stay; prolonged hospital LoS defined as ≥7 days; AUROC = area under the receiver operating characteristic curve; STUMBL = Study of the Management of Blunt Chest Wall Trauma.

Performance measures	Overall complications	90-day mortality	ICU admission	Prolonged hospital LoS	Rib fixation surgery
Cut-off STUMBL score	18.5	17.5	26.5	17.5	21.5
Accuracy	0.63	0.62	0.83	0.64	0.68
Sensitivity	0.63	0.49	0.83	0.57	0.67
Specificity	0.63	0.75	0.75	0.88	0.85
PPV	0.74	0.95	0.99	0.94	0.98
NPV	0.50	0.13	0.11	0.36	0.11
AUROC	0.63	0.62	0.79	0.73	0.76

## Discussion

The STUMBL score has been utilised in many A&E departments throughout the UK due to its reported high sensitivity, specificity, PPV, and NPV. Here, we elucidated the validity and utility of this risk stratification tool in predicting complications following blunt thoracic trauma.

This study investigated the utility of the STUMBL prognostic scoring system in patients with isolated blunt thoracic trauma admitted to a major trauma centre in England. To our knowledge, this is the first study to be conducted in a major trauma centre in England on the external validation of the STUMBL score. The creation of the scoring tool and first external validation by Battle and colleagues [19] was in Wales, and further validations by other authors were conducted in New Zealand [21], Scotland [22], Italy [23], and Australia [24]. Table 7 shows the sensitivity, specificity, PPV, and NPV of the STUMBL score that have been reported by authors.

**Table 7:** Validation values reported from different studies on the predictive ability of the STUMBL score in pulmonary complications. * = if STUMBL score ≥11; # = if STUMBL score >12; † = if STUMBL score >17. STUMBL = Study of the Management of Blunt Chest Wall Trauma; PPV = positive predictive value; NPV = negative predictive value; AUROC = area under the receiver operating characteristic curve; NR = not reported.

Studies measuring the validity of the STUMBL score	Sensitivity	Specificity	PPV	NPV	AUROC
Battle and colleagues^*^ [19]	80%	96%	93%	86%	0.96
Mukerji and colleagues^#^ [21]	61.7%	72.4%	NR	NR	0.73
Callisto and colleagues^* ^[22]	79%	77.9%	41.9%	94.9%	0.84
Giamello and colleagues^*^ [23]	95%	64%	50%	97%	0.90
Webb and colleagues^†^ [24]	62%	67.3%	21.1%	92.6%	0.76

The median STUMBL score of those discharged was similar in both the groups who did not develop complications and those who did develop complications (14.5 and 14, respectively) (Figure 4). Again, similar scores were produced in the admitted population regardless of complication development (no complications: 22, complications: 23). However, Giamello and colleagues [23] showed different median scores for those discharged with no complications at a score of 8 and those admitted with no complications with a median score of 16, whilst showing comparable median scores in patients who were discharged and developed complications (13) and those who were admitted and developed complications (21). This perhaps demonstrates further variables that may have been identified and considered by the assessing physician to determine hospital admission, which are not currently included within the scoring system.

The probability of developing a complication increased with a rising STUMBL score, as seen in Table 4. These figures are like Battle and colleagues’ [19] findings in their first validation study and Callisto and colleagues’ [22] study, with more complications seen up to a STUMBL score of 26-30. However, a score of 31+ showed fewer complications than the 26-30 group in this study, whilst both Battle and colleagues [19] and Callisto and colleagues [22] showed an increase in complications even with the 31+ group. Using the cohorts 0-11, 12-26, and ≥27, as recommended by Battle and colleagues [19], the results showed that overall complications increased with a rising score, with an increasing hospital and ICU admission with each cohort (Figure 4). The components of the overall complications were further analysed, with each component increasing in likelihood with an increasing score (Figure 5). A STUMBL score of ≥27 was found to be a major predictor for needing rib fixation surgery, which has not yet been reported in the literature.

The STUMBL cut-off score of >11 was used, as this was the threshold recommended by Battle and colleagues [19] to admit patients, as the probability of complications rises significantly after this score. The sensitivity of the threshold score of >11 was found to be lower in this study compared to that of Battle and colleagues [19] (25% and 80%, respectively). On assessment of the specificity (88%), PPV (78%), and NPV (41%), these performance measures were lower than those reported in the original study (specificity = 96%, PPV = 93%, and NPV = 86%). The specificity of this cut-off score was the lowest among all other validation studies of the STUMBL score (Table 7).

Physicians’ judgement in predicting complications and therefore admitting patients was also assessed. The sensitivity (65.0%) of a physician’s own judgement in predicting complications was lower than a STUMBL score of >11, but specificity (66.2%), PPV (53.3%), and NPV (76.2%) were considerably higher than a STUMBL score of >11. This suggests that a physician’s own judgement is more effective at predicting complications than a STUMBL score of >11.

This study yielded an AUROC score of 0.57 when the STUMBL score was >11 in predicting overall complications following rib fractures (Figure 6). This fell short of the recommended AUROC of 0.8 for accepting the cut-off as a strong model [28] and was significantly different from the originally reported AUROC of 0.96 in Battle and colleagues' study [19]. Mukerji and others [21] also found similar results, reporting an AUROC of 0.73; Buchholz and colleagues [29] reported a score of 0.65, and Webb and colleagues [24] reported a score of 0.76. However, Giamello and others [23] reported an acceptable AUROC at 0.90. One must consider that the AUROC values reported by the authors varied in their cut-off scores of predicting overall complications following rib fractures (Table 7). When the AUROC score was assessed for the individual components of the overall outcomes, the score was poor at predicting 90-day mortality (AUROC = 0.56), LRTI (AUROC = 0.58), prolonged LoS (AUROC = 0.62), and ICU admission (AUROC = 0.60) (Figure 6). However, Mukerji and colleagues [21] found that a score of >12 was acceptable in predicting mortality (AUROC = 0.92) and prolonged LoS (AUROC = 0.80).

Within the five previous validation studies, the optimal cut-off STUMBL score for predicting all complications varied from ≥11 in Battle and colleagues’ [19] original study to >26 in Callisto and colleagues’ [22] study. Webb and authors [24], Mukerji and colleagues [21], and Giamello and authors [23] found scores of 17.5, >12, and 16, respectively. The optimal cut-off score established in this study for predicting overall complications was 18.5 (Table 6). Battle and colleagues [19] recommended that if patients score ≥27, they should be admitted to the ICU. Mukerji and colleagues [21] and Giamello and colleagues [23] recommended cut-off scores of >18 and 24 for ICU admission, respectively. This study found a similar cut-off score of 26.5 for ICU admission (Table 6). These differing cut-off scores highlight a significant flaw in Battle and colleagues’ [19] original recommendations of >12 for predicting complications and ≥27 for admission to the ICU.

When further analysis was done on the individual components of the primary outcome, this study found a recommended cut-off score of 17.5 to predict 90-day mortality and 17.5 prolonged LoS (Table 6). Mukerji and colleagues [21] recommend a cut-off score of >12 to predict 90-day mortality. These authors and Webb and colleagues [24] both reported that a score of >15 and 18.5, respectively, should be used to predict prolonged LoS.

There are several possible reasons why the STUMBL score performed less well in this study than in Battle and colleagues’ [19] original study. First, the patient samples in each study differ in chronic lung disease, cardiac disease, smoking status, and pre-injury anticoagulation, with the patients in the original study having more comorbidities. However, age, number of fractures, and respiratory rate were similar in both cohorts. The original study also did not report other patient demographics [19]. However, subsequent validation studies reported similar baseline characteristics with age, female sex, falls as a mechanism of injury, smoking status, and pre-injury anticoagulation with this study [21-24].

Second, the definitions of some parameters in the STUMBL score were not clear. Battle and colleagues [19] did not define chronic lung disease. Consequently, this study used a very broad definition of this condition, encompassing COPD, asthma, ILD, emphysema, and pulmonary fibrosis. Additionally, the current study characterised LRTI differently from the original study, which relied solely on inpatient note documentation of pulmonary complications. Instead, we utilised imaging, patient note documentation, and GP records to identify complications, as this method proved to be more sensitive. Furthermore, the lower SpO2 targets for COPD patients were not considered in the original STUMBL score, nor was pre-hospital treatment with supplemental oxygen considered, meaning that SpO2 on arrival may not be accurate. We believe that these variables should be addressed in the future, should the STUMBL score be refined.

Third, it is important to consider that both the design and inception of the prognostic score and the first external validation study were conducted by Battle and colleagues [19]. Since this score’s development, all authors have reported reduced sensitivity, specificity, PPV, and NPV compared to the original study, including our current study (Table 7) [19,21-24].

Finally, Callisto and colleagues [22] report that patients with polytrauma, such as head, abdominal, limb, spinal, and pelvic injuries, were included in their patient sample. However, all other studies, including Battle and colleagues’ [19] original study, do not specify whether patients with only blunt thoracic trauma were included or if the sample also included patients with polytrauma [21-24]. Our interpretation of isolated blunt thoracic trauma meant rib fractures only, with no associated external thoracic injury, thus excluding 294 patients from our original sample. We believed that including the polytrauma patients may skew the results, as this may mean longer LoS and an increase in overall complication development, which may have affected Callisto and colleagues’ [22] complication and mortality rates, as well as the accuracy of the score’s sensitivity, specificity, PPV, and NPV. Further clarification is needed by Battle and colleagues [19] to see if the score can be used in polytrauma patients.

Interestingly, Buchholz and colleagues [29] have adapted the STUMBL score by removing age as a variable and replacing it with chest tube insertion, pulmonary contusions on CT scan imaging, and Glasgow Coma Scale score, as the authors found that age was a poor predictor of complications after rib fractures. Whether the score can be further adapted to include polytrauma patients, along with these other variables and those identified in the literature that increase the morbidity and mortality of rib fractures, such as the position of the fracture on the rib, could form the basis for further study in this research field [30]. Additionally, its use could be further adapted for other purposes, such as predicting the need for rib fixation surgery or analgesia management after rib fractures to prevent re-admissions for chest pain.

Limitations

This is a single-centre retrospective cohort study, which inherently carries limitations that may lead to a risk of bias, suggesting that the generalisability of results should be regarded with caution. Given that the data have been collected from a single centre, there is limited generalisability of these results. One must also consider that this research did not adjust for confounding variables, as the principal aim was to assess the validity of the existing STUMBL score. Not adjusting for confounding variables may limit the ability to determine whether associations observed are attributable to the STUMBL score itself rather than to other underlying factors, such as the mechanism of injury. Further research is required to assess if these additional factors significantly affect the predictability of these outcomes.

Furthermore, there was only one data extractor in this study, with no inter-investigator agreement or reliability. Although the researcher was blinded during data collection to calculate the overall STUMBL score, there remains a potential risk of measurement bias as incorrect data may have been recorded. Selection bias was minimised through stringent inclusion and exclusion criteria, along with an extensive list of ICD-10 codes utilised to identify eligible patients for this study. However, some patients may not have been included in this sample if they were incorrectly coded. With patients who were on supplemental oxygen, a score was designated for the SpO2 value, which was not included in the original study. This may have led to incorrect score calculations and may have underestimated or overestimated the STUMBL score. Potential miscalculations of the STUMBL score due to the ambiguity of some of the STUMBL variables, as described above, and misclassification of outcomes due to inconsistent documentation must be considered. As a result of these limitations, the conclusions of our study should be considered with care.

## Conclusions

The morbidity and mortality results following traumatic rib fractures presenting at a major trauma centre are in keeping with previously published findings: 90-day mortality was 9.3% and overall morbidity was 37.2%. Our single-centre study suggests that the recommended STUMBL risk-stratification tool commonly used in A&E departments in the UK to guide hospital admission following traumatic rib fractures may be suboptimal in predicting complications at a cut-off score of >11 and therefore may have limited use in its clinical utility in its current form. However, a score of 18.5 performed sufficiently in predicting overall complications, a score of 17.5 predicted 90-day mortality, 17.5 predicted prolonged LoS, and 26.5 predicted ICU admission. In our opinion, the STUMBL score requires recalibration and adaptation to optimise its function in assisting clinicians’ decision-making to optimise the care of this cohort of patients and the efficiency of A&E departments.

## Appendices

Comparison of the performance of the A&E clinician decision (decision to admit) with a STUMBL score of >11 for predicting complications

**Table 8:** Baseline characteristics of patients who were admitted and discharged based on A&E clinicians' judgements. Statistically significant associations are indicated in bold text. A&E = accident and emergency; SD = standard deviation; IQR = interquartile range; RTA = road traffic accident; SpO2 = oxygen saturation; O2 = oxygen; AF = atrial fibrillation; HTN = hypertension; IVDU = intravenous drug user; RA = rheumatoid arthritis; OA = osteoarthritis; VTE = venous thromboembolism; LoS = length of stay; ICU = intensive care unit; LRTI = lower respiratory tract infection; KW = Kruskal-Wallis test; F = Fisher’s exact test; MW = Mann-Whitney U-test; NA = not applicable; STUMBL = Study of the Management of Blunt Chest Wall Trauma.

		Discharged (n = 239)	Hospital admission (n = 199)			Test statistic	P-value
			No ICU admission (n = 187)		ICU admission (n = 12)		
Age, mean (SD) or median (IQR) (years)		62.5 ± 17.9	76 (61.5-83.5)		60.9 ± 21.2	34.13 (KW)	3.879x10^-8^
Number of ribs fractured, mean (SD) or median (IQR)		2 (1-3)	4 (3-6)		7.2 (2.6)	105.96 (KW)	<2.2x10^-16^
Male sex, n (%)		156 (65.3%)	120 (64.2%)		6 (50.0%)	NA (F)	0.54
Smoking status, n (%)							
Non-smoker		116 (48.5%)	82 (43.9%)		4 (33.3%)	NA (F)	0.45
Ex-smoker		77 (32.2%)	68 (36.4%)		6 (50.0%)	NA (F)	0.33
Current smoker		46 (19.2%)	36 (19.3%)		2 (16.7%)	NA (F)	1.00
Mechanism of injury, n (%)							
Fall from height		37 (15.5%)	18 (9.6%)		1 (8.3%)	NA (F)	0.16
Fall from standing		101 (42.3%)	88 (47.1%)		3 (25.0%)	NA (F)	0.26
Fall down stairs		31 (13.0%)	30 (16.0%)		1 (8.3%)	NA (F)	0.64
Fall from sitting		12 (5.0%)	7 (3.7%)		0 (0.0%)	NA (F)	0.79
Direct chest trauma		7 (2.9%)	3 (1.6%)		0 (0.0%)	NA (F)	0.64
Kicked by an animal		1 (0.4%)	3 (1.6%)		0 (0.0%)	NA (F)	0.40
Assault		5 (2.1%)	9 (4.8%)		0 (0.0%)	NA (F)	0.28
Sporting accident		3 (1.3%)	2 (1.1%)		0 (0.0%)	NA (F)	1.00
RTA - car		14 (5.9%)	11 (5.9%)		4 (33.3%)	NA (F)	0.01
RTA - motorbike		7 (2.9%)	9 (4.8%)		1 (8.3%)	NA (F)	0.25
RTA - bicycle		5 (2.1%)	4 (2.1%)		1 (8.3%)	NA (F)	0.33
RTA - pedestrian		2 (0.8%)	1 (0.5%)		1 (8.3%)	NA (F)	0.14
Other		14 (5.8%)	2 (1.0%)		0 (0.0%)	NA (F)	0.03
SpO_2 _(%), n (%)							
SpO_2_ 95-100		215 (90.0%)	121 (64.7%)		4 (33.3%)	NA (F)	2.53x10^-12^
SpO_2_ 90-94		18 (7.5%)	23 (12.3%)		2 (16.7%)	NA (F)	0.13
SpO_2_ 85-89		1 (0.4%)	6 (3.2%)		1 (8.3%)	NA (F)	0.02
SpO_2_ 80-84		0 (0.0%)	1 (0.5%)		0 (0.0%)	NA (F)	0.45
SpO_2_ 75-79		0 (0.0%)	0 (0.0%)		0 (0.0%)	NA (F)	1.00
SpO_2_ 70-74		1 (0.4%)	0 (0.0%)		0 (0.0%)	NA (F)	1.00
SpO_2_ on supplemental O_2_		4 (1.7%)	36 (19.3%)		5 (41.7%)	NA (F)	9.488x10^-12^
Comorbidities, n (%)							
Pre-injury anticoagulation		31 (13.0%)	34 (18.2%)		0 (0.0%)	NA (F)	0.12
Chronic lung disease		66 (27.6%)	67 (35.8%)		5 (41.7%)	NA (F)	0.14
Cardiac disease		60 (25.1%)	61 (32.6%)		2 (16.7%)	NA (F)	0.16
Hypercholesterolaemia/hyperlipidaemia		55 (23.0%)	50 (26.7%)		3 (25.0%)	NA (F)	0.66
Type 2 diabetes		37 (15.5%)	33 (17.6%)		0 (0.0%)	NA (F)	0.32
AF		24 (10.0%)	26 (13.9%)		0 (0.0%)	NA (F)	0.26
HTN		95 (39.7%)	88 (47.1%)		4 (33.3%)	NA (F)	0.26
History of kidney disease		33 (13.8%)	29 (15.5%)		0 (0.0%)	NA (F)	0.40
History of cancer		26 (10.9%)	31 (16.6%)		2 (16.7%)	NA (F)	0.20
History of stroke		22 (9.2%)	23 (12.3%)		2 (16.7%)	NA (F)	0.36
Current or ex-IVDU		2 (0.8%)	4 (2.1%)		1 (8.3%)	NA (F)	0.12
RA		5 (2.1%)	3 (1.6%)		1 (8.3%)	NA (F)	0.29
OA		46 (19.2%)	44 (23.5%)		2 (16.7%)	NA (F)	0.55
History of alcohol excess		16 (6.7%)	21 (11.2%)		1 (8.3%)	NA (F)	0.21
History of VTE		11 (4.6%)	15 (8.0%)		1 (8.3%)	NA (F)	0.25
Dementia		10 (4.2%)	23 (12.3%)		0 (0.0%)	NA (F)	0.0056
Parkinson’s disease		4 (1.7%)	8 (4.3%)		0 (0.0%)	NA (F)	0.28
STUMBL score, mean (SD) or median (IQR)		14 (10-18)	23.2 (7.7)		30.9 (10.2)	120.42 (KW)	<2.2x10^-16^
Hospital LoS, mean (±SD) or median (IQR) (days)		-	8.7 ± 9.1		15.1 (11.1)	5.30 (MW)	0.02
Admission speciality, n (%)							
Medicine		-	94 (50.3%)		0 (0.0%)	NA (F)	0.0004
Surgery		-	93 (49.7%)		12 (100.0%)	NA (F)	0.0004
ICU LoS, mean ± SD (days)		-	-		8.5 ± 10.8	NA (F)	NA
Complications, n (%)							
Overall complications		57 (23.8%)	100 (53.5%)		6 (50.0%)	NA (F)	6.493x10^-10^
LRTI		18 (7.5%)	66 (35.3%)		3 (25.0%)	NA (F)	1.745x10^-12^
Pneumothorax		1 (0.4%)	7 (3.7%)		0 (0.0%)	NA (F)	0.045
Haemothorax		1 (0.4%)	9 (4.8%)		0 (0.0%)	NA (F)	0.01
Pulmonary contusion		1 (0.4%)	4 (2.1%)		0 (0.0%)	NA (F)	0.28
Rib fixation surgery		1 (0.4%)	13 (7.0%)		6 (50.0%)	NA (F)	2.784x10^-9^
Re-admission		19 (7.9%)	7 (3.7%)		0 (0.0%)	NA (F)	0.16
90-day mortality		15 (6.3%)	24 (12.8%)		2 (16.7%)	NA (F)	0.03
Prolonged hospital stay		-	82 (43.9%)		7 (58.3%)	NA (F)	0.0056

STUMBL = 12-26 (n = 268)

**Table 9:** Baseline characteristics and outcomes of patients who suffered from rib fractures with a calculated STUMBL score of 12-26. Statistically significant associations are indicated in bold text. SD = standard deviation; IQR = interquartile range; RTA = road traffic accident; SpO2 = oxygen saturation; O2 = oxygen; AF = atrial fibrillation; HTN = hypertension; IVDU = intravenous drug user; RA = rheumatoid arthritis; OA = osteoarthritis; VTE = venous thromboembolism; LoS = length of stay; ICU = intensive care unit; LRTI = lower respiratory tract infection; KW = Kruskal-Wallis test; F = Fisher’s exact test; MW = Mann-Whitney U-test; NA = not applicable; STUMBL = Study of the Management of Blunt Chest Wall Trauma.

	Discharged (n = 142)	Hospital admission (n = 126)		Test statistic	P-value
		No ICU admission (n = 123)	ICU admission (n = 3)		
Age, mean (SD) (years)	68.3 (15.2)	72.0 (15.7)	39.0 (18.7)	10.872 (ANOVA)	0.00049
Number of ribs fractured, mean (SD) or median (IQR)	2 (2-3)	3 (2-5)	4.7 (0.6)	31.897 (KW)	1.185x10^-7^
Male sex, n (%)	93 (65.5%)	78 (63.4%)	2 (66.7%)	NA (F)	0.68
Smoking status, n (%)					
Non-smoker	62 (43.7%)	57 (46.3%)	1 (33.3%)	NA (F)	0.88
Ex-smoker	51 (35.9%)	43 (35.0%)	0 (0.0%)	NA (F)	0.66
Current smoker	29 (20.4%)	23 (18.7%)	2 (66.7%)	NA (F)	0.15
Mechanism of injury, n (%)					
Fall from height	24 (16.9%)	14 (11.4%)	0 (0.0%)	NA (F)	0.38
Fall from standing	60 (42.3%)	57 (46.3%)	1 (33.3%)	NA (F)	0.81
Fall down stairs	21 (14.8%)	21 (17.1%)	0 (0.0%)	NA (F)	0.77
Fall from sitting	9 (6.3%)	5 (4.1%)	0 (0.0%)	NA (F)	0.65
Direct chest trauma	4 (2.8%)	2 (1.6%)	0 (0.0%)	NA (F)	0.71
Kicked by an animal	1 (0.7%)	3 (2.4%)	0 (0.0%)	NA (F)	0.37
Assault	0 (0.0%)	7 (5.7%)	0 (0.0%)	NA (F)	0.008
Sporting accident	0 (0.0%)	1 (0.8%)	0 (0.0%)	NA (F)	0.47
RTA - car	6 (4.2%)	5 (4.1%)	1 (33.3%)	NA (F)	0.19
RTA - motorbike	5 (3.5%)	3 (2.4%)	0 (0.0%)	NA (F)	0.75
RTA - bicycle	3 (2.1%)	3 (2.4%)	1 (33.3%)	NA (F)	0.09
RTA - pedestrian	1 (0.7%)	1 (0.8%)	0 (0.0%)	NA (F)	1.00
Other	8 (5.6%)	1 (0.8%)	0 (0.0%)	NA (F)	0.11
SpO_2_ (%), n (%)					
SpO_2_ 95-100	123 (86.6%)	85 (69.1%)	1 (33.3%)	NA (F)	0.0003
SpO_2_ 90-94	14 (9.9%)	15 (12.2%)	0 (0.0%)	NA (F)	0.69
SpO_2_ 85-89	1 (0.7%)	2 (1.6%)	1 (33.3%)	NA (F)	0.026
SpO_2_ 80-84	0 (0.0%)	1 (0.8%)	0 (0.0%)	NA (F)	0.47
SpO_2_ 75-79	0 (0.0%)	0 (0.0%)	0 (0.0%)	NA (F)	1.00
SpO_2_ 70-74	1 (0.7%)	0 (0.0%)	0 (0.0%)	NA (F)	1.00
SpO_2_ on O_2_	2 (1.4%)	20 (16.3%)	1 (33.3%)	NA (F)	5.061x10^-6^
Comorbidities, n (%)					
Pre-injury anticoagulation	24 (16.9%)	20 (16.3%)	0 (0.0%)	NA (F)	1.00
Chronic lung disease	52 (36.6%)	36 (29.3%)	0 (0.0%)	NA (F)	0.24
Cardiac disease	50 (35.2%)	44 (35.8%)	0 (0.0%)	NA (F)	0.69
Hypercholesterolaemia/hyperlipidaemia	42 (29.6%)	32 (26.0%)	0 (0.0%)	NA (F)	0.59
Type 2 diabetes	27 (19.0%)	21 (17.1%)	0 (0.0%)	NA (F)	0.86
AF	19 (13.4%)	13 (10.6%)	0 (0.0%)	NA (F)	0.71
HTN	71 (50.0%)	55 (44.7%)	0 (0.0%)	NA (F)	0.25
History of kidney disease	27 (19.0%)	16 (13.0%)	0 (0.0%)	NA (F)	0.38
History of cancer	19 (13.4%)	23 (18.7%)	0 (0.0%)	NA (F)	0.42
History of stroke	18 (12.7%)	16 (13.0%)	0 (0.0%)	NA (F)	1.00
Current or ex-IVDU	2 (1.4%)	3 (2.4%)	1 (33.3%)	NA (F)	0.053
RA	2 (1.4%)	3 (2.4%)	0 (0.0%)	NA (F)	0.68
OA	37 (26.1%)	34 (27.6%)	0 (0.0%)	NA (F)	0.73
History of alcohol excess	9 (6.3%)	17 (13.8%)	1 (33.3%)	NA (F)	0.052
History of VTE	7 (4.9%)	7 (5.7%)	0 (0.0%)	NA (F)	0.82
Dementia	8 (5.6%)	14 (11.4%)	0 (0.0%)	NA (F)	0.25
Parkinson’s disease	2 (1.4%)	3 (2.4%)	0 (0.0%)	NA (F)	0.68
Hospital LoS, mean (SD) or median (IQR) (days)	-	5.1 (2.1-10.5)	6.2 (0.8)	0.10494 (MW)	0.77
Admission speciality, n (%)					
Medicine	-	64 (52.0%)	0 (0.0%)	NA (F)	0.12
ICU LoS, mean ± SD (days)	-	-	2.3 ± 1.5	NA	NA
Complications, n (%)					
Overall complications	33 (23.2%)	61 (49.6%)	2 (66.7%)	NA (F)	9.588x10^-6^
LRTI	8 (5.6%)	40 (32.5%)	1 (33.3%)	NA (F)	1.411x10^-8^
Pneumothorax	1 (0.7%)	3 (2.4%)	0 (0.0%)	NA (F)	0.37
Haemothorax	0 (0.0%)	5 (4.1%)	0 (0.0%)	NA (F)	0.0543
Pulmonary contusion	1 (0.7%)	3 (2.4%)	0 (0.0%)	NA (F)	0.37
Rib fixation surgery	0 (0.0%)	6 (4.9%)	1 (33.3%)	NA (F)	0.002
Re-admission	10 (7.0%)	5 (4.1%)	0 (0.0%)	NA (F)	0.52
90-day mortality	9 (6.3%)	12 (9.8%)	0 (0.0%)	NA (F)	0.50
Prolonged hospital stay	-	49 (39.8%)	0 (0.0%)	NA (F)	0.28

STUMBL ≥27 (n = 82)

**Table 10:** Baseline characteristics and outcomes of patients who suffered from rib fractures with a calculated STUMBL score of ≥27. Statistically significant associations are indicated in bold text. SD = standard deviation; IQR = interquartile range; RTA = road traffic accident; SpO2 = oxygen saturation; O2 = oxygen; AF = atrial fibrillation; HTN = hypertension; IVDU = intravenous drug user; RA = rheumatoid arthritis; OA = osteoarthritis; VTE = venous thromboembolism; LoS = length of stay; ICU = intensive care unit; LRTI = lower respiratory tract infection; KW = Kruskal-Wallis test; F = Fisher’s exact test; MW = Mann-Whitney U-test; NA = not applicable; STUMBL = Study of the Management of Blunt Chest Wall Trauma.

	Discharged (n = 18)	Hospital admission (n = 64)		Test statistic	P-value
		No ICU admission (n = 55)	ICU admission (n = 9)		
Age, mean (SD) or median (IQR) (years)	77.0 (66.8-80.1)	75.9 (12.1)	68.2 (17.0)	2.4145 (KW)	0.23
Number of ribs fractured, mean (SD)	6.5 (1.5)	6.7 (2.0)	8.0 (2.4)	2.083 (ANOVA)	0.13
Male sex, n (%)	9 (50.0%)	36 (65.5%)	4 (44.4%)	NA (F)	0.31
Smoking status, n (%)					
Non-smoker	8 (44.4%)	20 (36.4%)	3 (33.3%)	NA (F)	0.78
Ex-smoker	8 (4.4%)	24 (43.6%)	6 (66.7%)	NA (F)	0.49
Current smoker	2 (11.1%)	10 (18.2%)	0 (0.0%)	NA (F)	0.45
Mechanism of injury, n (%)					
Fall from height	0 (0.0%)	4 (7.3%)	1 (11.1%)	NA (F)	0.34
Fall from standing	13 (72.2%)	29 (52.7%)	2 (22.2%)	NA (F)	0.052
Fall down stairs	1 (5.6%)	7 (12.7%)	1 (11.1%)	NA (F)	0.86
Fall from sitting	1 (5.6%)	2 (3.6%)	0 (0.0%)	NA (F)	1.00
Direct chest trauma	0 (0.0%)	1 (1.8%)	0 (0.0%)	NA (F)	1.00
Kicked by an animal	0 (0.0%)	0 (0.0%)	0 (0.0%)	NA (F)	1.00
Assault	1 (5.6%)	1 (1.8%)	0 (0.0%)	NA (F)	0.55
Sporting accident	0 (0.0%)	0 (0.0%)	0 (0.0%)	NA (F)	1.00
RTA - car	1 (5.6%)	4 (7.3%)	3 (33.3%)	NA (F)	0.055
RTA - motorbike	0 (0.0%)	5 (9.1%)	1 (11.1%)	NA (F)	0.41
RTA - bicycle	0 (0.0%)	1 (1.8%)	0 (0.0%)	NA (F)	1.00
RTA - pedestrian	0 (0.0%)	0 (0.0%)	1 (11.1%)	NA (F)	0.11
Other	1 (5.6%)	1 (1.8%)	0 (0.0%)	NA (F)	0.55
SpO_2_ (%), n (%)					
SpO_2_ 95-100	12 (66.7%)	27 (49.1%)	3 (33.3%)	NA (F)	0.25
SpO_2_ 90-94	4 (22.2%)	8 (14.5%)	2 (22.2%)	NA (F)	0.59
SpO_2_ 85-89	0 (0.0%)	4 (7.3%)	0 (0.0%)	NA (F)	0.73
SpO_2_ 80-84	0 (0.0%)	0 (0.0%)	0 (0.0%)	NA (F)	1.00
SpO_2_ 75-79	0 (0.0%)	0 (0.0%)	0 (0.0%)	NA (F)	1.00
SpO_2_ 70-74	0 (0.0%)	0 (0.0%)	0 (0.0%)	NA (F)	1.00
SpO_2_ on O_2_	2 (11.1%)	16 (29.1%)	4 (44.4%)	NA (F)	0.17
Comorbidities, n (%)					
Pre-injury anticoagulation	7 (38.9%)	14 (25.5%)	0 (0.0%)	NA (F)	0.10
Chronic lung disease	11 (61.1%)	30 (55.5%)	5 (55.6%)	NA (F)	0.94
Cardiac disease	6 (33.3%)	16 (29.1%)	2 (22.2%)	NA (F)	0.87
Hypercholesterolaemia/hyperlipidaemia	5 (27.8%)	17 (30.9%)	3 (33.3%)	NA (F)	1.00
Type 2 diabetes	4 (22.2%)	11 (20.0%)	0 (0.0%)	NA (F)	0.38
AF	5 (27.8%)	11 (20.0%)	0 (0.0%)	NA (F)	0.27
HTN	12 (66.7%)	31 (56.4%)	4 (44.4%)	NA (F)	0.58
History of kidney disease	2 (11.1%)	13 (23.6%)	0 (0.0%)	NA (F)	0.20
History of cancer	1 (5.6%)	7 (12.7%)	2 (22.2%)	NA (F)	0.44
History of stroke	3 (16.7%)	7 (12.7%)	2 (22.2%)	NA (F)	0.71
Current or ex-IVDU	0 (0.0%)	1 (1.8%)	0 (0.0%)	NA (F)	1.00
RA	0 (0.0%)	0 (0.0%)	1 (11.1%)	NA (F)	0.11
OA	0 (0.0%)	10 (18.2%)	2 (22.2%)	NA (F)	0.08
History of alcohol excess	2 (11.1%)	4 (7.3%)	0 (0.0%)	NA (F)	0.66
History of VTE	3 (16.7%)	8 (14.5%)	1 (11.1%)	NA (F)	1.00
Dementia	1 (5.6%)	9 (16.4%)	0 (0.0%)	NA (F)	0.38
Parkinson’s disease	1 (5.6%)	5 (9.1%)	0 (0.0%)	NA (F)	1.00
Hospital LoS, mean (SD) or median (IQR) (days)	-	8.00 (4.5-12.9)	18.1 (11.4)	3.4954 (MW)	0.06
Admission speciality, n (%)					
Medicine	-	26 (47.3%)	0 (0.0%)	NA (F)	0.008
ICU LoS, mean ± SD (days)	-	-	10.5 (11.9)	NA	NA
Complications, n (%)					
Overall complications	7 (38.9%)	37 (67.3%)	4 (44.4%)	NA (F)	0.07
LRTI	4 (22.2%)	25 (45.5%)	2 (22.2%)	NA (F)	0.12
Pneumothorax	0 (0.0%)	4 (7.3%)	0 (0.0%)	NA (F)	0.73
Haemothorax	0 (0.0%)	4 (7.3%)	0 (0.0%)	NA (F)	0.73
Pulmonary contusion	0 (0.0%)	1 (1.8%)	0 (0.0%)	NA (F)	1.00
Rib fixation surgery	1 (5.6%)	7 (12.7%)	5 (55.6%)	NA (F)	0.0056
Re-admission	2 (11.1%)	2 (3.6%)	0 (0.0%)	NA (F)	0.26
90-day mortality	3 (16.7%)	11 (20.0%)	2 (22.2%)	NA (F)	1.00
Prolonged hospital stay	-	33 (60.0%)	7 (77.8%)	NA (F)	0.47
